# The Origin of Carbonate Veins Within the Sedimentary Cover and Igneous Rocks of the Cocos Ridge: Results From IODP Hole U1414A

**DOI:** 10.1029/2018GC007729

**Published:** 2018-10-11

**Authors:** Jennifer Brandstätter, Walter Kurz, Sylvain Richoz, Matthew J. Cooper, Damon A. H. Teagle

**Affiliations:** ^1^ Institute of Earth Sciences, NAWI Graz Geocenter University of Graz Graz Austria; ^2^ Department of Geology Lund University Lund Sweden; ^3^ Ocean and Earth Science, National Oceanography Centre Southampton University of Southampton Southampton England, UK

**Keywords:** Cocos Ridge, IODP, carbonate veins, isotope geochemistry, elemental composition, fluid‐rock interaction

## Abstract

Carbonate veins in the igneous basement and in the lithified sedimentary cover of the Cocos Ridge at International Ocean Discovery Program (IODP) Hole 344‐U1414A reveal the hydrologic system and fluid‐rock interactions. IODP Hole 344‐U1414A was drilled on the northern flank of the Cocos Ridge and is situated 1 km seaward from the Middle America Trench offshore Costa Rica. Isotopic and elemental compositions were analyzed to constrain the fluid source of the carbonate veins and to reveal the thermal history of Hole 344‐U1414A. The formation temperatures (oxygen isotope thermometer) of the carbonate veins in the lithified sedimentary rocks range from 70 to 92 °C and in the basalt from 32 to 82 °C. ^87^Sr/^86^Sr ratios of the veins in the altered Cocos Ridge basalt range between 0.707307 and 0.708729. The higher ratios are similar to seawater strontium ratios in the Neogene. ^87^Sr/^86^Sr ratios lower 0.7084 indicate exchange of Sr with the igneous host rock. The calcite veins hosted by the sedimentary rocks are showing more primitive ^87^Sr/^86^Sr ratios <0.706396. The isotopic compositions indicate seawater, modified into a hydrothermal fluid by subsequent heating, as the main fluid source. Low‐temperature alteration and the presence of a high‐temperature fluid resulted in different carbonate precipitates forming up to several cm thick veins. The geochemical data combined with age data of the sedimentary rocks suggest intraplate seamount volcanism in the area between the Galapagos hot spot and the Cocos Island as an additional heating source, after the formation of the Cocos Ridge at the Galapagos hot spot.

## Key Points

- Oxygen and strontium isotope ratios of carbonate veins suggest seawater and hydrothermally modified seawater as fluid source
- Isotopic and elemental compositions reveal fluid‐rock interactions in the Cocos Ridge basalt and in the lithified sedimentary rocks
- Intraplate seamount volcanism in the area between the Galapagos hot spot and the Cocos Island acted as an additional heating source

## 1 Introduction

**Figure 1:**
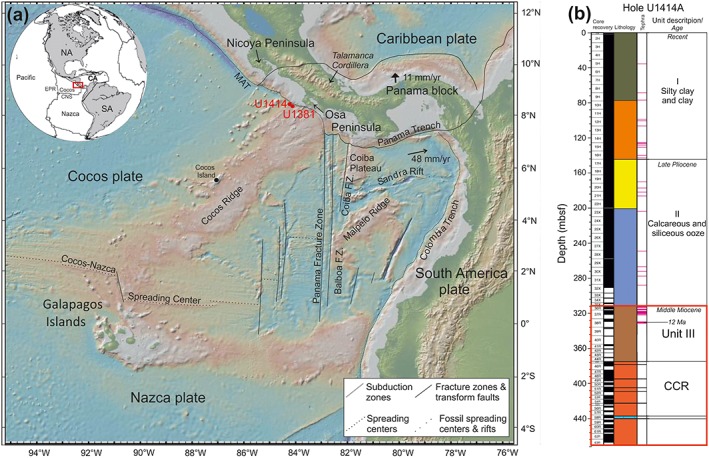
(a) Digital elevation map of the Costa Rica area (http://www.geomapapp.org; Ryan et al., 2009) showing the location of the Costa Rica Seismogenesis Project (CRISP) drilling area with drilling Site U1414, and the context of the general plate tectonic setting. F.Z., fracture zone; MAT‐Middle America Trench. Inset shows plate tectonic setting of Central America and the surrounding plates North America (NA), Caribbean (CA), South America (SA), and the CRISP drilling area (red frame). EPR, East Pacific Rise, CNS, Cocos‐Nazca spreading center. (b) Stratigraphy of Hole U1414A, comprising three sedimentary units and eight igneous basement units with one intercalated sedimentary layer (after Harris et al., 2013b).

At the erosive plate boundary offshore Costa Rica the Cocos Plate is being subducted under the Caribbean Plate and the highstanding aseismic Cocos Ridge (CCR) has lifted the seismogenic zone into the accessible reach of scientific oceanic drilling. Drilling on the subducting and overriding plate was achieved as part of the Costa Rica Seismogenesis Project (CRISP) during International Ocean Discovery Program (IODP) Expedition 344. CRISP has the main objective to clarify processes that control nucleation and seismic rupture of large earthquakes at erosional subduction zones (Harris et al., 2013a). A particular target is how the hydrogeochemical processes within the subduction zone affect seismicity and chemical alteration (Solomon et al., 2009). Circulating and trapped fluids within the sediments and ocean crust of the incoming plate can be incorporated into the subduction system, enter the subduction zone and may be released into fracture systems within the overriding plate. The understanding of the impact of these fluids on the material in the subduction zone is also essential for a better understanding of the seismogenesis in subduction zones. Therefore, vein‐hosting sedimentary and igneous rock material from IODP Hole U1414A, located on the incoming Cocos Plate 1 km seaward from the Middle America Trench offshore the western margin of Costa Rica (Figure 1a), was investigated. The results from IODP Hole U1414A describe the hydrologic system and identify fluid pathways and fluid‐rock interactions within the upper crustal sections of the oceanic Cocos Plate (Harris et al., 2013b).

The sedimentary and igneous rocks of IODP Hole U1414A are transected by various hydrothermal veins that are mainly filled by carbonates. These hydrothermal veins host inclusions of low to seawater‐like salinity fluids (salinity from 0 to 3.4 mass% NaCl, except vesicles with ca. 5 mass% NaCl) trapped over a wide temperature range from 40 to approximately 400 °C. These fluid inclusions reveal multiple fluid entrapment stages suggesting that fluid flow occurred over several episodes (Brandstätter et al., 2016).

Hydrothermal fluid flow in the uppermost oceanic crust is generally characterized by penetration of cold seawater into the porous, permeable igneous rocks. This seawater heats up with depth and evolves into hydrothermal fluids that become buoyant and ascend toward the seafloor as hot vent fluids (Alt, 1995). This circulation can result in strong fluid‐rock exchange causing major chemical changes to the host basalts and influence the chemistry of the seawater (e.g., Alt & Teagle, 1999; Coggon et al., 2004, 2010; Staudigel et al., 1981, 1996). Evidence of such fluid circulations in the upper oceanic crust is provided by hydrothermal veins and by mineralogical changes within the host basalts. Veins can give important information about the source and composition of the migrating fluids and fluid circulation and offer the opportunity to reveal the thermal history, past fluid circulation and fluid‐rock interactions (e.g., Blyth et al., 2000; Burkhard & Kerrich, 1988; Dietrich et al., 1983; Drake & Tullborg, 2009; Rye & Bradbury, 1988). Vein minerals in both the igneous basement rocks and the overlying lithified sedimentary rocks sampled from IODP Hole U1414A allow the reconstruction of element ratios in the fluids from which they are precipitated (Coggon et al., 2004, 2010).

In this study, we present analyses of the elemental and isotopic composition of calcite and aragonite veins (CCV) and of the surrounding host rocks to unravel the thermal and fluid history at IODP Hole 344‐U1414A and to clarify the temperature variations derived from fluid inclusions. Oxygen and strontium isotopic compositions of CCV provide useful constraints on fluid sources and reflect the extent of the chemical exchange between seawater and the host rocks of the upper oceanic crust (Bach et al., 2011; Coggon et al., 2004; Muehlenbachs & Clayton, 1976). We used ^87^Sr/^86^Sr ratios, oxygen, carbon isotopic, and elemental compositions, particularly the rare earth elements and Yttrium (REY), to define the fluid source and pathways of paleofluids through the sedimentary cover and the igneous basement, and to reveal fluid‐rock interaction processes.

## 2 Geological Setting and Sample Description

The investigated CCV were sampled in drill cores recovered from IODP Hole U1414A, located 1 km seaward of the Middle America Trench offshore Costa Rica, where the Cocos Plate, that previously formed at the Cocos‐Nazca spreading center (also known as Galapagos spreading center), subducts beneath the Caribbean Plate (Figure 1a). This region is a seismically active area with a history of Mw > 7 (Mw = moment magnitude scale) earthquakes (Harris et al., 2013a). The complex history of the Cocos Plate, formed at the Cocos‐Nazca spreading center (also known as Galapagos spreading center), is dominated by the interaction of the Galapagos hot spot and the Cocos‐Nazca spreading center resulting in the formation of the highstanding NE‐SW trending aseismic CCR (Barckhausen et al., 2001; Brandstätter et al., 2016; Harpp et al., 2005; Hoernle et al., 2002; Meschede & Barckhausen, 2000). The NE end of the tholeiitic CCR was dated at 13.0–14.5 Ma (Werner et al., 1999) and tephra layers within soft sediment layers from Site U1381, about 11 km southeast from Hole U1414A, record Plinian eruptions from 16.5 to 8 Ma (Schindlbeck et al., 2015). A younger bathymetric features in this area are the ~2 Ma alkalic Cocos Island volcanoes and an adjacent group of seamounts (Figure 1a) that form the Cocos Island Province (Harpp et al., 2005; Schindlbeck, Kutterolf, Freundt, Andrews, et al., 2016; Werner et al., 2003).

IODP Hole U1414A was drilled during IODP Expedition 344 (CRISP 2A) and serves as a reference site to constrain the lithostratigraphy and the hydrologic system of the subducting CCR. The recovered cores of Hole U1414A comprise 375.50 m of sediment and 96.35 m of igneous basement (Harris et al., 2013a). The sedimentary sequence is subdivided in three sedimentary units (Unit I‐III), where Units I and II are composed of soft sediments and Unit III is characterized by strongly lithified calcareous and siliceous siltstone to sandstone (Harris et al., 2013a; Figure 1b). Shipboard analyzed nannofossil and radiolaria assemblages constrain the age of Unit I from the Recent to Early Pleistocene with continuous sedimentation to the Middle Miocene (Unit II; Sandoval et al., 2017). Ar/Ar dating of tephras in Unit III yields an age of ~12 Ma (344‐U1414A‐38‐R, 76–78 cm; Schindlbeck, Kutterolf, Freundt, Alvarado et al., 2016). The accumulation rate varied on the incoming Cocos Plate through the past. The Neogene sedimentation rates were around 13–43 m/Myr offshore Nicoya and 14–21 m/Myr offshore Osa Peninsula. In the Early Pleistocene accumulation rates were around 5 m/Myr. Closer to the trench late Pleistocene hemipelagic sedimentation rates increased to 200 m/Myr at Site U1414 and 150 m/Myr at Site U1381 (Schindlbeck, Kutterolf, Freundt, Straub, et al., 2016). The presence of a Late Miocene to Pliocene sediment hiatus (1.5 to 8 Ma) at Site U1381 is in strong contrast to the continuous sedimentation of the closely located Site U1414 and could be the result of temporally and regionally interplays of morphology, erosion, and biogenic productivity (Schindlbeck, Kutterolf, Freundt, Straub, et al., 2016).

The igneous basement broadly comprises aphyric to highly phyric massive and thin basaltic flows. The abundance of phenocrysts, such as plagioclase, clinopyroxene, and olivine, varies. The basalts are slightly to strongly hydrothermally altered, characterized by brecciation, partial replacement of groundmass mineralogy, partial to complete replacement of phenocrysts, particularly olivine and clinopyroxene, and veins, some with alteration halos. There is a lack of oxidative secondary minerals, such us iron‐oxyhydroxide and celadonite (Harris et al., 2013b). Vesicle abundance varies throughout the different igneous basement units, and vesicles are partially to completely infilled by secondary minerals, particularly saponite and smectite, and fibrous calcite and quartz (Harris et al., 2013b).

Hydrothermal veins are distributed within the lithified sedimentary rocks of Unit III and the igneous basement. In the lithified sedimentary rocks, the veins are mainly filled by blocky, coarse calcite, crosscutting the rarely appearing fibrous radial quartz veins. Noticeable are wall rock fragments embedded within the vein filling.

The vein filling in the basalt consists of clay minerals (smectite and saponite), calcium carbonates, quartz, and pyrite. All four minerals occur in centimeter‐thick veins, in some cases with several generations of calcium carbonate. Pyrite is present in veins and in both host rocks. Veins in the basalt are more or less surrounded by a brown selvage of predominantly clay minerals resulting from basalt alteration. The veins in the basalt occur within different growth morphologies: syntaxial, antitaxial, and composite veins (Brandstätter et al., 2016). Syntaxial veins are mainly composed of blocky or coarse aragonite, antitaxial veins solely of fibrous calcite. Composite veins show spherulitic blocky quartz in the center, sometimes overgrown by small calcite grains. Quartz is surrounded by fibrous and/or blocky calcite and a clay selvage (Brandstätter et al., 2017).

The CCV hosted by the sedimentary units and the CCR basalt are described as CCV‐S and CCV‐B, respectively, in the following chapters.

## 3 Methods‐Analytical Techniques

The selection of samples for the isotopic and elemental composition analyses were based on microscopic thin section observations, fluid inclusion data (Brandstätter et al., 2016), and aim to represent the entire sequence of the recovered drill core. Sedimentary and basaltic host rock samples were drilled manually with a hand‐held carbonite‐tipped drill, and CCV were drilled with a computer‐controlled micromill‐sampler (ESI New Wave, drilling head 100 μm). Visibly altered portions and heterogeneous areas of the veins were avoided. Whole rock samples of the host rocks, the extracted carbonate phase of the powder of the bulk sedimentary host rock, and the CCV from both rock types were analyzed for strontium isotopic and elemental composition in the Isotope Geochemistry Laboratories of the National Oceanography Centre Southampton, University of Southampton. Stable isotopic compositions of the CCV in both rock types were determined at the Institute of Earth Sciences, NAWI Graz Geocenter, University of Graz, and at the Institute of Water Resources Management, Hydrogeology and Geophysics, Joanneum Research, Graz. Additionally strontium isotopes of thin calcite veins in the basalt were analyzed at the NAWI Graz Central Lab for Water, Minerals and Rocks, Graz University of Technology.

### 3.1 Stable Isotope (δ^13^C, δ^18^O) Analyses

Sample powders of 9 CCV‐S from the sedimentary cover and 11 CCV‐B (8 calcite and 3 aragonite) from the basalt were reacted with 100% phosphoric acid at 70 °C in a Kiel II automated reaction system. The evolved carbon dioxide gas was analyzed with a Finnigan Delta Plus mass spectrometer at the Institute of Earth Sciences, NAWI Graz Geocenter, University of Graz. The δ^13^C and δ^18^O values are corrected according to the NBS19 standard and are reported in per mill (‰) relative to the Vienna‐PeeDee Belemnite (V‐PDB) standard. For each sample two to five measurements were carried out with an analytical precision of 1σ < 0.05‰ for δ^13^C, < 0.1‰ for δ^18^O. Additionally, δ^13^C and δ^18^O values of four sedimentary host rock powder samples and three CCV‐S and two CCV‐B were measured by a Finnigan DELTAplusXP mass spectrometer at the Institute of Water Resources Management, Hydrogeology and Geophysics, Joanneum Research, Graz. The overall error of reproducibility is <0.1‰ (VPDB) for both δ^13^C and δ^18^O values. Formation temperatures of calcite and aragonite are calculated assuming equilibrium with seawater (0‰ Vienna SMOW standard mean ocean water [VSMOW]), using the fraction equations of Kim and O'Neil (1997) for calcite and Kim et al. (2007) for aragonite.

### 3.2 Strontium Isotopic and Elemental Composition

Ten CCV‐S from sedimentary Unit III and seven CCV‐B (five calcite and two aragonite) from the basalt, the corresponding host rocks as well as the extracted carbonate phase of the siliceous and calcareous cemented sedimentary host rock, were analyzed for elemental composition and strontium isotopes in the Isotope Geochemistry Laboratories of the National Oceanography Centre Southampton, University of Southampton. Carbonate samples were dissolved in 5% acetic acid, the carbonate phases were leached from the sedimentary host rocks with 5% acetic acid, and the whole rocks were digested using HF/HNO_3_. Trace and selected major elements were measured by inductively coupled plasma‐mass spectrometry using a Thermo Scientific X‐Series II. Samples and standards were spiked with internal standard elements and corrected for interferences and blank and then calibrated using a suite of international rock standards (JB‐3, JB‐1a, JGb‐1, BHVO‐2, and BIR‐1, reference data from GeoReM database; Jochum et al. 2005). Long‐term accuracy relative to reference values is 3–5%. Sr was isolated with 80 μl Sr‐Spec columns and eluted with 3 M HNO_3_. The dried down Sr fraction was loaded onto a single Ta filament with a Ta activator solution and analyzed on a Thermo Scientific Triton Thermal Ionization Mass Spectrometer, using a static procedure with amplifier rotation (on a ^88^Sr beam of 2 V) for 300 ratios. Fractionation was corrected using an exponential correction normalized to ^86^Sr/^88^Sr = 0.1194. NIST 987 was run as a reference standard and the long‐term average (150 analyses) on this instrument is 0.710245 ± 0.000025 (2SD).

Additionally five samples, two fibrous calcite vesicles, and three veins with fibrous calcite and blocky quartz were dissolved by dilute (2%) HNO_3_ to avoid dissolution of clay minerals. Sr was separated using Sr‐specific extraction chromatographic resin (Eichrom) columns. About 5 ml of 3 M HNO_3_ were used for element elution and 1 ml 0.1 M HNO_3_ for Sr collection. Strontium isotopic ratios were determined on a Nu Instruments Plasma II multicollector‐inductively coupled plasma‐mass spectrometry at the NAWI Graz Central Lab for Water, Minerals and Rocks, Graz University of Technology. NIST SRM 987 yielded ^87^Sr/^86^Sr ratios of 0.710253 ± 0.000067 (2σ; *n* = 12) and all samples were corrected relative to the value of 0.710250 for NIST SRM 987.

## 4 Results

**Figure 2:**
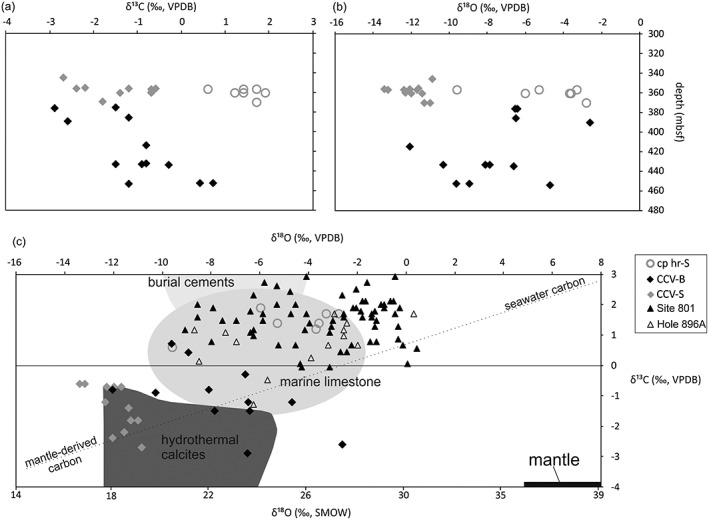
Diagrams of (a) carbon and (b) oxygen isotopic composition of the calcite and aragonite veins (CCV) in the sedimentary rocks (grey) and in the basalt (black) and the calcareous sedimentary host rock (open circles) versus depth (meters below seafloor). (c) Oxygen isotopic versus carbon isotopic composition of additionally CCV data of ODP Site 801 (black triangle, Alt & Teagle, 2003) and ODP Hole 896A (open triangle, Teagle et al., 1996). The dashed line shows mixing between mantle‐derived carbon and seawater carbon (Stakes & O'Neil, 1982) and in black the field of the isotopic composition of the mantle (Pineau & Javoy, 1983). Fields of isotopic compositions of carbonates derived from Nelson and Smith (1996) and Rollinson (1993).

The carbon sources can be constrained by the carbon isotopic composition of the calcite, reflecting the δ^13^C of dissolved CO_2_ (Ohmoto & Rye, 1979). The oxygen isotopic composition of the vein calcite relates to the δ^18^O of the water from which it precipitated and the temperature‐dependent oxygen isotope fractionation between calcium carbonate and the fluid (Kim & O'Neil, 1997; Urey, 1947; Zheng, 1999). Fields of typical marine limestones, burial cements, and hydrothermal calcites and a mixing line of seawater carbon and mantle‐derived carbon are plotted in Figure 2c (Nelson & Smith, 1996; Rollinson, 1993). The ^87^Sr/^86^Sr ratio of the calcium carbonate veins records the Sr‐isotopic composition of the fluids from which they precipitate, that in turn reflects the composition of contemporaneous seawater, pore fluid compositions, and fluid‐host rock Sr exchange (e.g., Coggon et al., 2004, 2010). The chemical evolution of the fluids from which the CCV were precipitated is reflected by changes in the trace element concentrations (Coggon et al., 2004, 2010).

### 4.1 Isotopic Composition

#### 4.1.1 Carbon and Oxygen Isotopes

Carbon isotopes of the CCV‐S and CCV‐B vary between −2.7 and −0.6‰ and −2.9 and 0.7‰, respectively, with a slight increase of δ^13^C with depth in the basalt (Table 1 and Figure 2a). The aragonite veins in the basalt have relatively low δ^13^C values from −2.9 to −1.5‰. The higher values are typical for modern seawater (around 0‰), whereby the lower values indicate low‐temperature interaction of seawater or pore fluids with the basalt (e.g., Alt & Teagle, 2003). The δ^18^O values of the CCV‐S cluster in a narrow range (17 to 19.7‰ SMOW), in contrast to the CCV‐B that show a much wider range of values from 18.5 to 28.2‰ SMOW, indicating various formation temperatures of CCV‐B (calcite and aragonite; Figure 2b). The stable isotopic values of the corresponding sedimentary host rock lie between 0.6 and 1.9‰ for carbon isotopes and between 21.0 and 28.0‰ SMOW for the oxygen isotopes (Table 1). In Figure 2c carbon and oxygen isotope fields for burial cements, marine limestones, and hydrothermal calcites (Nelson & Smith, 1996; Rollinson, 1993) are represented. CCV in oceanic crust from Site 801 and Hole 896A, the calcareous sedimentary host rock, and the majority of the CCV‐B plot in the field of marine limestones, whereas the CCV‐S are mainly in the field of hydrothermal calcites.

**Table 1:** Isotopic Composition of the CCV and Two Vesicles

Sample number	Depth (m below seafloor)	Type	Mineral	δ^13^C_CCV_ (‰)_VPDB_	δ^18^O_CCV_ (‰)_VPDB_	δ^18^O_CCV_ (‰)_VSMOW_	T (°C)	δ^13^C_hr_ (‰)_VPDB_	δ^18^O_hr_ (‰)_VSMOW_
Unit III									
40R‐1‐W 6/9	345.6	V	C	−2.7	−10.9	19.7	74		
41R‐1‐W 8/10	355.3	V	C	−2.2	−11.6	19	79		
41R‐2‐W 12/13	356	V	C	−2.4	−12.1	18.5	82.2		
41R‐2‐W 57/63	356.5	V	C	−0.6	−13.4	17	92		
41R‐2‐W 69/72	356.7	V	C	−0.6	−13.2	17.3	90.3	0.6	21
41R‐2‐W 73/77	356.7	V	C	−1.2	−12.4	18.2	84.4	1.4	25.5
41R‐2‐W 93/99	356.9	V	C	−0.7	−11.7	18.9	79.5	1.7	27.5
42R‐1‐W 13/15	360.2	V	C	−0.7	−12.3	18.2	84	1.4	27.2
42R‐1‐W 31/36	360.4	V	C	−0.7	−12	18.5	81.8	1.9	24.7
42R‐1‐W 39/40	360.5	V	C	−1.4	−11.4	19.2	70.2	1.2	27.1
44R‐1‐W 12/14	369.9	V	C	−1.8	−11	19.6	74.6	1.7	28
44R‐1‐W 21/24	370.1	V	C	−1.8	−11.3	19.3	76.7		
CCR basalt									
45R‐2‐W 3/5	376	V	A	−1.5	−6.4	24.3	52.9		
46R‐2‐W 32/34	376.3	V	A	−2.9	−6.5	24.2	53.3		
47R‐2‐W 36/39	386.1	v	C	−1.2	−6.5	24.2	46.8		
48R‐1‐W 88/92	390	V	A	−2.6	−2.6	28.2	31.6		
53R‐1‐W 126/131	414.5	V	C + Q	−0.8	−12.1	18.5	82.3		
57R‐1‐W 38/43	433.2	V	C + Q	−0.8	−8.1	22.6	56.3		
57R‐1‐W 71/76	433.5	V	C	−1.5	−7.9	22.8	54.8		
57R‐1‐W 86/90	433.7	V	C + Q	−0.9	−10.3	20.3	70.2		
57R‐2‐W 46/52	434.5	V	C	−0.3	−6.6	24.1	47.7		
61R‐1‐W 45/49	452.6	V	C	0.7	−9.6	21	65.9		
61R‐1‐W 62/69	452.7	V	C	0.4	−8.9	21.7	61.5		
61R‐1‐W 62/69	452.7	V	C						
61R‐2‐W 101/109	454	v	C	−1.2	−4.7	26.1	36.7		

*Note*. hr, host rock; V, vein; v, vesicle; C, calcite; A, aragonite; Q, quartz.

#### 4.1.2 Strontium Isotopes

**Figure 3:**
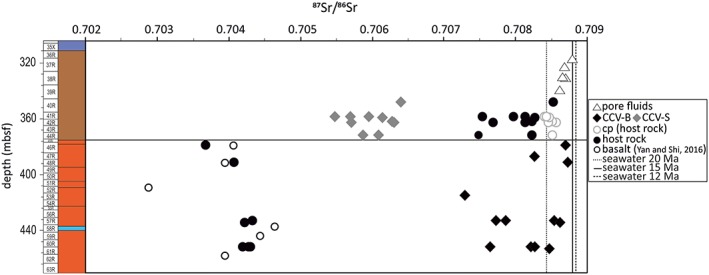
Strontium isotopic ratios of shipboard measured pore fluids (black rhombs), calcite and aragonite veins (CCV) in the sedimentary rocks (grey diamonds) and in the basalt (black diamonds), both host rock samples (black circles), and the carbonate phase of the sedimentary host rock (cp; open circles) versus depth (meters below seafloor). ^87^Sr/^86^Sr ratios of the CCR Ridge from Hole U1414A from Yan and Shi (2016; open black circles). The black line represents the boundary between sedimentary rocks and underlying basalt within the drillcore.

Strontium isotopes derived from shipboard measured pore fluids within the upper poorly indurated part of Unit III show a decrease of ^87^Sr/^86^Sr ratios with depth, from 0.70884 at 308 m below seafloor to 0.70861 at the deepest measureable sample (Figure 3 and Table 2). Intense carbonate recrystallization and cementation inhibited the liberation of pore fluids below 337 m below seafloor (Ross et al., 2015).

**Table 2:** Strontium Isotopic Ratios of CCV, Two Vesicles, the Corresponding Host Rocks (hr), and the Carbonate Phase of the Sedimentary Host Rock (cp‐hr)

Sample number	Type	^87^Sr/^86^Sr CCV	±2SE (×10^−6^)	^87^Sr/^86^Sr hr	±2SE (×10^−6^)	^87^Sr/^86^Sr cp‐hr	±2SE (×10^−6^)
Unit III							
40R‐1‐W 6/9	V	0.706396	15	0.708517	18		
41R‐1‐W 8/10	V	0.705691	16	0.707964	15	0.708427	18
41R‐2‐W 12/13	V	0.705484	17	0.707535	17	0.708403	15
41R‐2‐W 57/63	V	0.705959	14	0.70813	16		
41R‐2‐W 69/72	V	0.706141	15	0.708269	16	0.708455	14
41R‐2‐W 73/77	V	0.706283	16	0.708222	16	0.708504	16
41R‐2‐W 93/99	V	0.705715	16	0.707685	17	0.708456	14
42R‐1‐W 13/15	V	0.706304	14	0.708125	16	0.708561	15
42R‐1‐W 31/36	V	0.706094	16	0.708221	15	0.708506	16
42R‐1‐W 39/40	V	0.70587	15	0.707481	17		
CCR basalt							
46R‐2‐W 32/34	V	0.708687	15	0.703674	17		
47R‐2‐W 36/39^a^	v	0.708261	23				
48R‐1‐W 88/92	V	0.708729	13	0.704073	17		
53R‐1‐W 126/131^a^	V	0.707307	29				
57R‐1‐W 38/43^a^	V	0.707705	17				
57R‐1‐W 71/76	V	0.708533	16	0.704335	16		
57R‐1‐W 86/90^a^	V	0.707872	25				
57R‐2‐W 46/52	V	0.70862	16	0.704219	16		
61R‐1‐W 45/49	V	0.707641	15	0.704193	16		
61R‐1‐W 62/69	V	0.708313	15	0.704298	16		
61R‐1‐W 62/69	V	0.708215	15	0.704274	17		
61R‐2‐W 101/109^a^	v	0.708444	27				

*Note*. V, veins; v, vesicle; ^1^CCV measured at the NAWI Graz Central Lab for Water, Minerals and Rocks, Graz University of Technology. The other CCV and all host rock samples were measured at the Isotope Geochemistry Laboratories of the National Oceanography Centre Southampton, University of Southampton.

a Calcite and aragonite veins (CCV) measured at the NAWI Graz Central Lab for Water, Minerals and Rocks, Graz University of Technology. The other CCV and all host rock samples were measured at the Isotope Geochemistry Laboratories of the National Oceanography Centre Southampton, University of Southampton.

The analyzed vein material of sedimentary Unit III obtained ^87^Sr/^86^Sr ratios from 0.70548 to 0.70639 and the corresponding sedimentary host rocks from 0.707481 to 0.708517 (the extracted carbonate phase of the host rock from 0.708403 to 0.708561).

The CCR basalt yield typical ^87^Sr/^86^Sr ratios for altered basalt (Dasch et al., 1973); however, the basaltic host rock of the two aragonite samples shows lower ratios, in direction to fresh basalt. The CCV‐B yield ^87^Sr/^86^Sr ratios from 0.70729 to 0.70873 (Table 2). The measured ^87^Sr/^86^Sr ratios of the majority of the CCV‐B and the carbonate phase of Unit III are close to the range of the seawater composition during Miocene times (Figure 3). Sr isotopic composition of seawater increased from approximately 0.7088 in the Middle Miocene to approximately 0.70916 in recent times (McArthur et al., 2001). ^87^Sr/^86^Sr ratios of CCV‐B with quartz as additional mineral phase obtained lower ^87^Sr/^86^Sr ratios than syntaxial, blocky aragonite veins, and the fibrous calcite veins.

### 4.2 Elemental Composition

CCV, the corresponding host rocks and the carbonate phase of the sedimentary host rock, were analyzed for major elements (Ca, K, Fe, Mg, and Na) and trace elements (REE, Y, Mn, Li, Sr, Ba, Zn, Ni, Co, Cr, V, and Rb). Mg/Ca and Sr/Ca ratios from the CCV give indications about precipitation phases and the composition of the fluid from which the carbonates were precipitated, with due regard to the temperature of vein formation and the knowledge about the temperature dependence of element partitioning between mineral and fluid (Coggon et al., 2010).

REE + Y (REY) data of all carbonate rocks is presented in Post‐Archean Australian Shale (PAAS)‐normalized diagrams (Pourmand et al., 2012; Taylor & McLennan, 1985). Shale‐normalized (SN) elemental concentrations were used to calculate elemental anomalies. Different REY anomalies were used to constrain the fluid sources and the paleo‐redox conditions, normalized to PAAS (Pourmand et al., 2012). REY data of the CCR basalt are normalized to REE chondrite (Sun & McDonough, 1989). REEs occur in trivalent state and behave as a coherent group with a variation in chemical properties (e.g., Zhong & Mucci, 1995). Ce and Eu have the ability to adopt different oxidations states in aqueous solutions and can be used to constrain precipitation environments of carbonates (Bolhar et al., 2004; Bolhar & Van Kranendonk, 2007). Trivalent Ce can be oxidized to less soluble tetravalent Ce in oxygenated water; this fractionation leads to a negative Ce anomaly (Ce_SN_/Ce_SN_* = Ce_SN_/(Pr_SN_^2)/Nd_SN_; Lawrence et al., 2006; Tostevin et al., 2016) in modern well‐oxygenated seawater. Under extremely reducing conditions and higher temperatures, trivalent Eu can be reduced to soluble divalent Eu (eg., Bau, 1991; Danielson et al., 1992; Wang et al., 2014). Positive Eu anomalies (Eu_SN_/Eu_SN_* = 2*Eu_SN_/(Sm_SN_ + Gd_SN_); Lawrence et al., 2006; Tostevin et al., 2016) are typical for hydrothermal vent fluids, can be the result of plagioclase recrystallization reactions during magmatic rock alteration, and are mainly regarded as an indicator for hydrothermal input (e.g., Bolhar & Van Kranendonk, 2007; Wang et al., 2014; Wheat et al., 2002; Zhang et al., 2015). Positive La anomaly (La_SN_/La_SN_* = La_SN_/(Pr_SN_*(Pr_SN_/Nd_SN_)^2); Lawrence et al., 2006) and positive the Gd anomaly (Gd_SN_/Gd_SN_* = Gd_SN_/((2*Tb_SN_)‐Dy_SN_); Bolhar et al., 2004) are characteristic for modern seawater REY systematics. The Yttrium anomaly (=Y/Ho ratio) serves for the identification of the sedimentary origin and different water types/fluid sources (e.g., Bau & Dulski, 1999; Bolhar et al., 2004; Bolhar & Van Kranendonk, 2007; Wang et al., 2014; Zhang et al., 2015). The twin elements Y and Ho are geochemically similar but can differ in their complexation behavior and behave incoherently in marine environment due to lower stabilities of surface complexes of Y (e.g., Bau et al., 1997; Bolhar & Van Kranendonk, 2007; Pack et al., 2007). For modern oxygenated seawater the typical Y/Ho ratio is >45 and for marine carbonates 44–74 (Bau et al., 1996; Bolhar & Van Kranendonk, 2007). With increase of water depth and under anoxic conditions the molar ratio decreases, due to preferential sorption of Ho relative to Y on Fe and Mn oxyhydroxides (Bau & Dulski, 1999).

#### 4.2.1 Sedimentary Host Rock

Elemental composition and calculated anomalies of the strongly lithified calcareous and siliceous cemented sedimentary host rock and the extracted carbonate phase can be found in the electronic data supplement. The ratios of the major elements Sr/Ca and Mg/Ca of the carbonate phase yield mean values of 0.32 and 92.87 mmol/mol, respectively (Table 3). The Ce anomaly (Ce/Ce*) _SN_ show weak depleted values (mean values 0.83), the La anomaly (La/La*) _SN_ yields a positive mean value of 1.96, and the Eu anomaly (Eu/Eu*) _SN_ shows slight positive values (mean value of 1.26), the same for the Gd anomaly (Gd/Gd*)_SN_ with a mean value of 1.23. The Y/Ho values show a range from 33.9 to 39.1. The sedimentary host rock shows enrichment of HREE relative to LREE and MREE ((Pr/Yb)_SN_ = 0.31, (Sm/Yb)_SN_ = 0.45; Table 4).

**Table 3:** Elemental Composition and Mg/Ca and Sr/Ca Ratios of the CCV‐S and the CCV‐B

	Ca	Mg	Na	Sr	Mn	Fe	U	Sc	Zr	Hf	Th	Mg/Ca	Sr/Ca
Sample	ppm	ppm	ppm	ppm	ppm	ppm	ppb	ppb	ppb	ppb	ppb	mmol/mol	mmol/mol
**Unit III**													
40R‐1‐W 6/9	383700	3809	110	107	1299	384	118	274	2185	3414	433.6	16.4	0.13
41R‐2‐W 57/63	367300	2319	59	105	2014	41	43	330	986	1590	117.9	10.4	0.13
41R‐2‐W 73/77	315500	2332	102	72	1746	161	74	454	544	998	46.7	122	0.1
41R‐2‐W 93/99	396300	2654	35	105	1705	334	35	128	382	560	26.0	11.0	0.12
42R‐1‐W 13/15	394300	2125	54	101	2050	13	17	159	237	329	16.9	8.9	0.12
42R‐1‐W 31/36	393500	2142	135	96	1997	222	91	349	888	1142	22.7	9.0	0.11
42R‐1‐W 39/40	382400	2031	78	82	1515	63	41	189	265	386	9.7	8.8	0.10
44R‐1‐W 12/14	392600	2417	30	105	2040	25	37	702	167	161	6.9	10.1	0.12
44R‐1‐W 21/24	401100	2590	23	90	2134	28	28	277	125	172	5.1	10.6	0.10
CCR basalt													
46R‐2‐W 32/34	343200	1909	460	2904	167.6	6604	3	90	186	272	5.5	9.2	3.87
48R‐1‐W 88/92	390200	358.8	93	4541	38.63	340	0	87	141	191	2.2	1.5	5.32
57R‐1‐W 71/76	282300	8183	793	162	13220	5834	4	1667	166	301	6.4	47.8	0.26
57R‐2‐W 46/52	342600	4956	107	135	13920	5248	0	1194	80	177	1.4	23.8	0.18
61R‐1‐W 45/49	182300	9697	3823	154	9623	4743	17	6361	143	186	3.3	87.7	0.39
61R‐1‐W 62/69	217200	8483	2241	181	13390	6703	14	4600	148	236	3.8	64.4	0.38
61R‐1‐W 62/69	337200	6640	1233	91	12620	4790	4	9104	199	351	2.1	32.5	0.12

*Note*. Samples 46R‐2‐W 32/34 and 48R‐1‐W 88/92 are aragonite veins.

**Table 4:** PAAS‐Normalized Anomalies of the CCV‐S and the CCV‐B

Sample	Ce/Ce*	Eu/Eu*	Gd/Gd*	La/La*	Y/Ho
Unit III					
40R‐1‐W 6/9	0.57	1.68	0.68	1.87	40
41R‐2‐W 57/63	0.89	1.45	1.04	2.43	45.2
41R‐2‐W 73/77	0.87	1.54	1.03	2.59	46.3
41R‐2‐W 93/99	0.98	1.46	1	2.27	46
42R‐1‐W 13/15	0.94	1.45	1.34	2.95	55.5
42R‐1‐W 31/36	1.02	1.36	1.43	3.07	48.5
42R‐1‐W 39/40	1.09	1.18	1.48	3.61	55.5
44R‐1‐W 12/14	0.98	1.22	1.19	2.1	42.6
44R‐1‐W 21/24	0.97	1.36	1.26	2.51	51.1
CCR basalt					
46R‐2‐W 32/34	1.11	1.47	1.09	1.52	34
48R‐1‐W 88/92	1.12	1.18	1.07	1.52	33.7
57R‐1‐W 71/76	1.2	1.23	1.13	1.7	33.7
57R‐2‐W 46/52	1.19	1.2	1.12	1.64	32.6
61R‐1‐W 45/49	1.15	1.45	1.01	1.52	25.8
61R‐1‐W 62/69	1.15	1.44	1.05	1.55	28.7
61R‐1‐W 62/69	1.16	1.41	1.04	1.74	26.5

#### 4.2.2 CCV‐S

**Figure 4:**
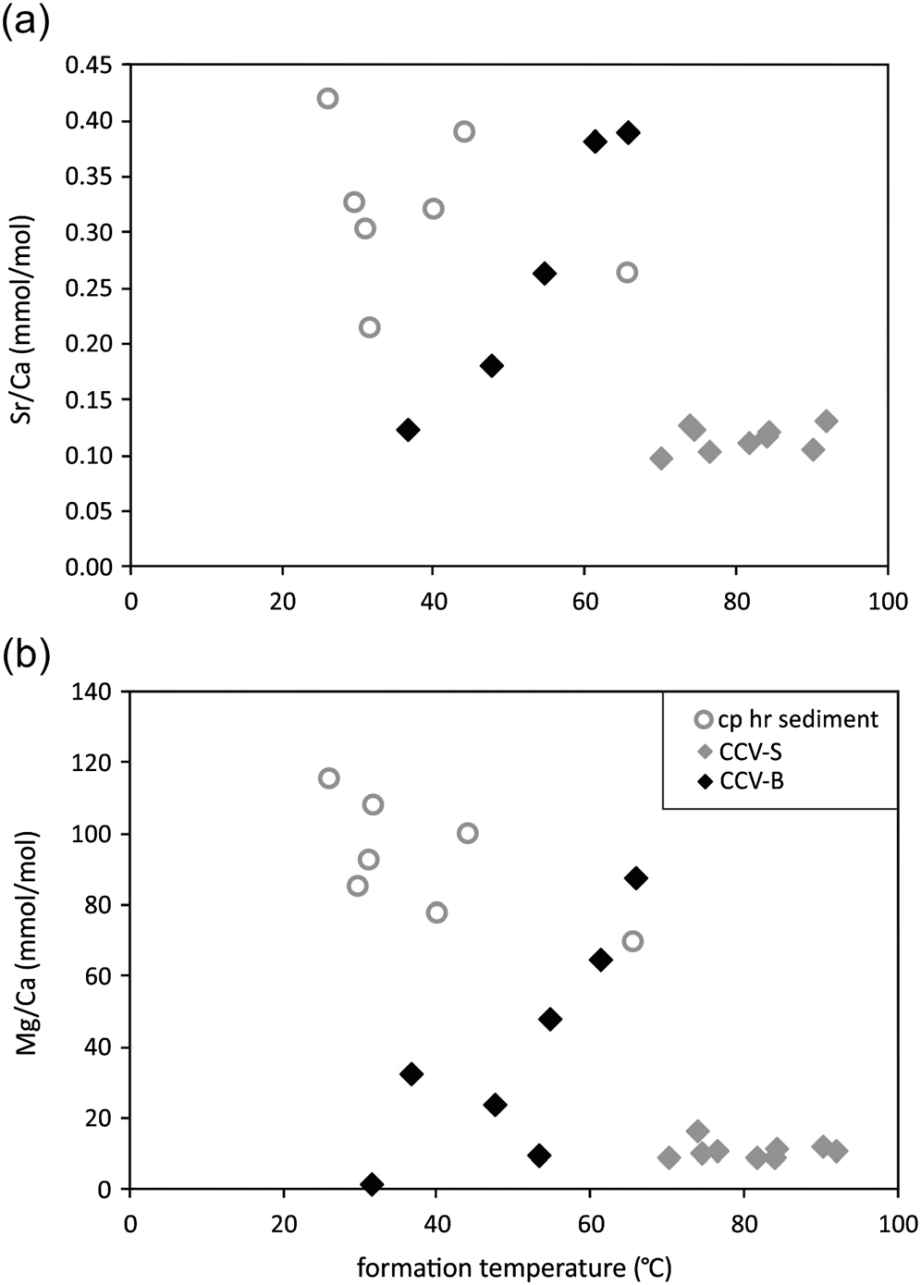
(a) Sr/Ca and (b) Mg/Ca in mmol/mol versus calculated formation temperature (using δ18O values, assuming equilibrium with seawater of 0‰ VSMOW) for the carbonate phase of the sedimentary host rock, CCV‐S and CCV‐B (aragonite veins with Sr/Ca of 3.9 and 5.3 mmol/mol and 53.5 and 32 °C, respectively, are excluded).

**Figure 5:**
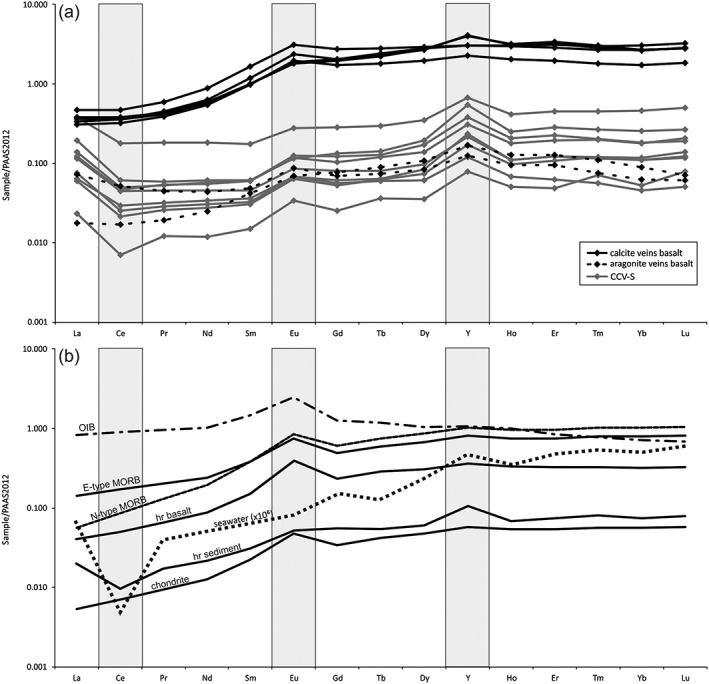
Selected REY patterns, normalized to PAAS of (a) CCV of Hole U1414A: calcite veins (black lines) and aragonite veins (dashed black lines) in the basalt, CCV in the sedimentary rocks (grey lines), and (b) of seawater (×10^6^; Jeandel et al., 2013), N‐type MORB, E‐type MORB, and OIB (dashed line; Sun & McDonough, 1989) and of the host rocks of the CCV‐S (hr sediment) and the CCV‐B (hr basalt). Important anomalies are highlighted in gray.

Ratios of the main elements Sr/Ca and Mg/Ca yield mean values of 0.12 and 10.83 mmol/mol, respectively (Figure 4 and Table 3). CCV transecting the sedimentary rocks of Unit III are characterized by a weak depletion of LREE to MREE (mean (Pr/Sm)_SN_ = 0.91) and more strongly depleted LREE and MREE relative to HREE (mean (Pr/Yb)_SN_ = 0.34; mean (Sm/Yb)_SN_ = 0.37), with insignificant negative Ce anomalies (mean (Ce/Ce*)_SN_ = 0.92). The PAAS‐normalized REY‐patterns of the CCV‐S show similarities with the sedimentary host rock and seawater (Figures 5a and 5b). The CCV‐S display pronounced a positive La anomaly (mean (La/La*)_SN_ = 2.60), slight positive Eu anomaly (mean (Eu/Eu*)_SN_ = 1.41), and weak Gd anomaly (mean (Gd/Gd*)_SN_ = 1.16. Y/Ho ratios cluster at a mean value of 47.85 (Table 4). Further elements show no distinctive trend or distribution, except of Zr (124 to 2185 ppb) and U (28 to 117 ppb).

#### 4.2.3 CCV‐B.

The CCV‐B can be subdivided into two groups, based on whether their main carbonate filling is aragonite or calcite. The aragonite veins, occurring in the upper part of the igneous sections, show rather high Sr/Ca ratios (3.87 to 5.32 mmol/mol) compared to calcium veins (mean value of 0.27 mmol/mol). The ratios of Mg/Ca ratios of the calcite veins are higher (51.25 mmol/mol) than the ratios for the aragonite veins (10.65 mmol/mol; Table 3). The Mg concentration in the calcite veins within the basalt shows a broader distribution compared to CCV‐S (Figure 4).

The CCV‐B show a strong to moderate depletion of LREE and MREE relative to HREE (mean (Pr/Yb)_SN_ = 0.26; mean (Sm/Yb)_SN_ = 0.51), similar to E‐type MORB and the igneous host rock (Figure 5). The CCV are characterized by a weak positive Ce anomaly (mean (Ce/Ce*)_SN_ = 1.16), more pronounced positive La anomaly (mean (La/La*)_SN_ = 1.60), slight positive Eu anomaly (mean (Eu/Eu*)_SN_ = 1.34) and also weak Gd anomaly (mean (Gd/Gd*)_SN_ = 1.06 (Table. 4). Y/Ho ratios show chondritic values, with a mean of 30.73. Certain elements (Sc, Th, V, and Co) have broader ranges and higher concentrations than the CCV in the sedimentary rocks.

The Sr concentrations of all vein samples are in average ~113 ppm (range 70 to 182 ppm, *n* = 14), except of two aragonite veins with much higher values of 2904 to 4541 ppm.

The CCV‐B and CCV‐S are clearly distinguishable by their manganese concentration, as CCV‐B have a tenfold higher Mn concentration, except the aragonite veins. The U concentration ranges from 17 to 117 ppb for the CCV‐S, whereas the CCV‐B have lower concentrations in U (<1 to 17 ppb). The corresponding host rocks show similar results, the sedimentary host rocks have high concentrations of U (2.4 to 5.5 ppm), and the basalt shows lower U concentrations (0.1 to 0.3 ppm).

## 5 Discussion

### 5.1 Fluid Source of the CCV

**Figure 6:**
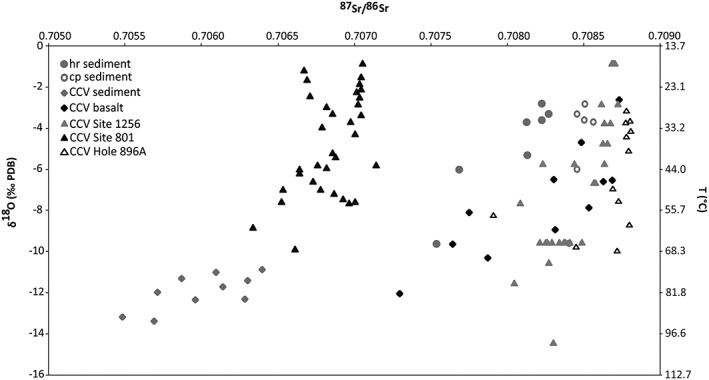
^87^Sr/^86^Sr ratios for CCV in the sedimentary rocks (grey rhombs), in the basalt (black rhombs), and the carbonate phase of the sedimentary host rock (grey open circles) versus oxygen isotopic composition and the corresponding formation temperature of calcite and approximate aragonite formation temperatures in equilibrium with water (δ^18^O 0‰ SMOW). Data from CCV from Site 1256 located on the Cocos Plate in 15 Ma crust (grey triangle; Coggon et al., 2010), in 170 Ma crust in the western Pacific from ODP Site 801 (black triangle; Alt & Teagle, 2003) and from Hole 896A in young, 6.8 Ma crust (Teagle et al., 1996).

The CCV‐S and CCV‐B can be clearly distinguished by their oxygen and strontium isotopic composition, except three *transitional* samples (from 53R‐1‐W 126/131 to 61R‐1‐W 45/49), which are composite CCV with quartz yielding similar oxygen isotope values like the CCV‐S and less radiogenic ^87^Sr/^86^Sr ratios than the other CCV‐B (Figure 6). A further CCV with quartz (sample 57R‐1‐W 38/43) indicates lower temperatures than the other transitional samples. This, however, could be attributed to the sample powder for the oxygen isotopic analysis, which was taken from the outer vein area, where fibrous calcite is intergrown with clay minerals. Formation temperatures (oxygen isotope thermometer) of a similar vein sample (53R‐1‐W 126/131), where the powder was taken from the vein center, are significantly higher. There is a positive correlation between the strontium ratios and the oxygen isotopic composition of the CCV in both rock types, that is, an increased temperature with decreased ^87^Sr/^86^Sr ratios (Figure 6). Less radiogenic strontium ratios results from seawater‐basalt and seawater‐sediments interaction and suggests higher formation temperatures for the CCV (Alt & Teagle, 2003).

Sr/Ca and Mg/Ca ratios of the CCV‐B trend to higher values with higher formation temperature and broader distribution, compared to CCV‐S, which could be attributed to multiple calcite precipitation phases within the basalt. The results of this study confirm the observation of Coggon et al. (2004) that calcites precipitated from a hotter fluid, tend to have rather basaltic, less radiogenic ^87^Sr/^86^Sr ratios, and lower Mg/Ca and Sr/Ca concentrations (Figures 4 and 6). The CCV‐S yield only limited ranges of Sr/Ca and Mg/Ca ratios for the whole temperature range, compared to the carbonate phase of the host sediments that have higher and more broadly distributed Sr/Ca and Mg/Ca values. The leached carbonate phase from the bulk sedimentary host rock is more heterogeneous, due to primary calcareous components and younger calcareous cements, and consists of more radiogenic strontium ratios than the bulk sedimentary host rock. This difference is presumably due to the silicate‐derived ^87^Sr, the existence of cryptic microveins and recrystallization in the bulk rock can be excluded as ^87^Sr source, due to the dissolution from the bulk sedimentary host rock.

The fluid source of the less radiogenic CCV‐S with higher formation temperatures could be a deeper sourced hot fluid that altered the basalt and penetrated the lithified sedimentary rocks of Unit III. The lower temperatures determined for the CCV‐B suggest that the first vein formation in the basalt was dominated by invaded seawater interacting with the cooling porous basalt.

The carbon isotopic values of the CCV yield a range from −3 to +0.7‰. The higher values are typical for marine carbonates (mean δ^13^C value around 0‰; Nelson & Smith, 1996) and the lower values indicate a fluid source more enriched in carbon, for example, from biogenic carbon and/or from a carbonic fluid derived from the altered CCR basalt. Magmatic carbon has a δ^13^C value around −5‰, mantle‐derived rocks have δ^13^C values in a range of −8 to −4‰ PDB (Pineau & Javoy, 1983), and vent fluids show values from −8 to −2‰ (Craig et al., 1980; Shilobreeva et al., 2011). Biogenic material, however, is rare; few fossils of radiolarians were found in Unit III (Sandoval et al., 2017). Compared with the results of Stakes and O'Neil (1982), the carbon isotopic values in this study plot within the field of calcite veins hosted by hydrothermally altered mid‐ocean‐ridge basalt.

CCV in basalts of ODP drill sites located on the Cocos Plate (Hole 896A) and Pacific Plate (Site 801) show generally positive δ^13^C values with a mean value of 0.6 and 2.1‰, respectively (Alt & Teagle, 2003; Teagle et al., 1996). The interaction of seawater with the basalt and the sedimentary host rocks is perceptible in the δ^13^C values and the ^87^Sr/^86^Sr ratios. Compared to the strontium isotope seawater curve (McArthur et al., 2001), the pore fluids from sample 38R‐2, 66–102 cm, indicate an age around 17 Ma, whereas the Ar/Ar dating of tephras from sample 38‐R, 76–78 cm yield an age of 12 Ma (Schindlbeck, Kutterolf, Freundt, Alvarado, et al., 2016). This suggests that the isotopic composition of the pore fluids was modified by fluid‐rock interaction. Correlation of the CCV‐B with the strontium isotope stratigraphy (McArthur et al., 2001) make sense for five samples, assuming that the maximum age of the CCR basalt of Hole U1414A is 20 Ma. One vesicle sample, two fibrous CCV‐B, and two aragonite samples show a decrease of age with decreasing depth. The CCV‐B with quartz as additional mineral phase are showing the opposite trend (Figure 3).

**Figure 7:**
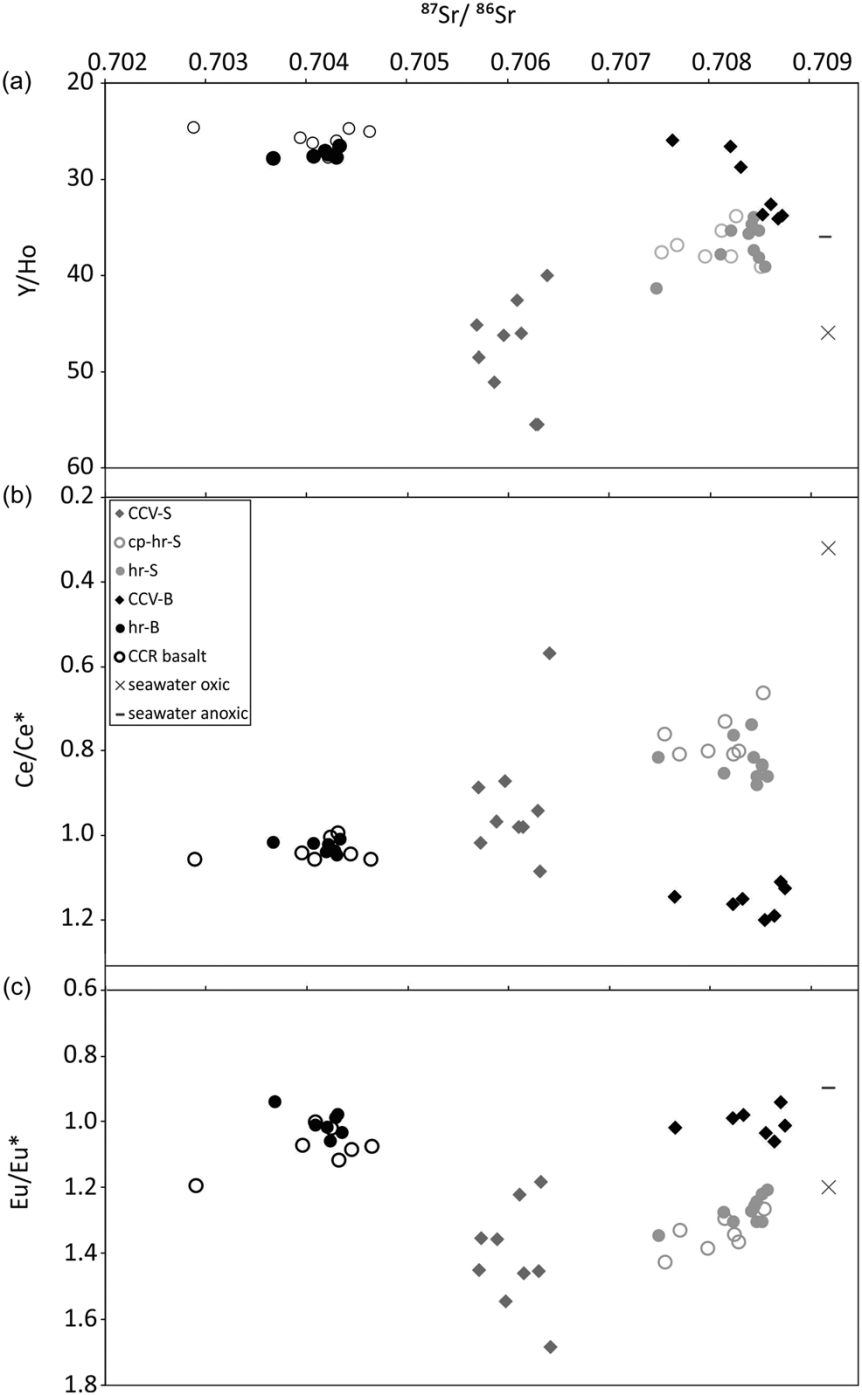
^87^Sr/^86^Sr ratios of CCV‐S (grey diamonds), carbonate phase of the sedimentary host rock (cp‐hr‐S, open grey circles) and the sedimentary host rock (hr‐S, grey circles), CCV‐B (black diamonds), igneous host rock (hr‐B, black circles) of this study, CCR basalt from Hole U1414A from Yan & Shi, 2016 (open black circles), and modern oxic (cross) and anoxic (line) seawater versus (a) Y/Ho ratio, (b) Ce/Ce* (normalized to PAAS) and (c) Eu/Eu* (normalized to PAAS).

To constrain the fluid source and fluid‐rock interaction, the calculated REY anomalies were plotted against the strontium isotopic composition to discriminate the CCV‐S from CCV‐B (Figure 7). The strontium isotopic composition of seawater was 0.7084 to approximately 0.7091 (McArthur et al., 2001) for the pertinent time period (20 Ma to recent), which includes the strontium isotope ratios of the carbonate phase of the sedimentary host rock and some CCV‐B samples. The CCV‐B with seawater like strontium isotopic composition tend to have also higher Y/Ho ratios (Figure 7a), whereas the less radiogenic CCV‐B plot in the field of Y/Ho ratios typical for basaltic rocks 26–33 (basalt reference material BCR‐1 [trachybasalt], BHVO‐1 [lava flow], BIR‐1 [tholeiite], and JB‐2, JB‐3, and GSR‐3 [flood basalt]; Dulski, 2001). Y/Ho ratios of the CCR basalt from IODP Hole U1414A range from 23 to 28 (supporting information). The less radiogenic ^87^Sr/^86^Sr and lower Y/Ho ratio veins indicate formation from fluids that had undergone more basalt hydrothermal exchange.

**Figure 8:**
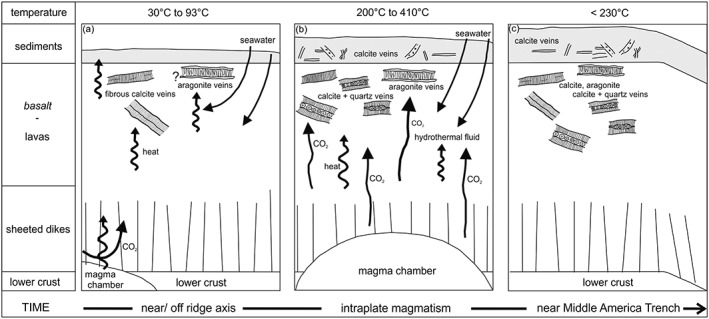
Schematic model of the evolution of the calcite and aragonite vein (CCV) scenarios (a) near or off ridge axis of the Cocos‐Nazca spreading center, (b) open‐marine setting with intraplate magmatism (Cocos Island) characterized by a shallow magma chamber, and (c) close to the Middle America Trench, not to scale. Temperature information derived from oxygen isotope thermometer, fluid inclusion analysis (Brandstätter et al., 2016), and deformation conditions (Brandstätter et al., 2017).

Modern seawater contains a large range of Y/Ho from 33 to 40 in near shore or restricted settings to 40–70 in open marine settings (Bau & Dulski, 1999; Bolhar & Van Kranendonk, 2007; Nozaki & Zhang, 1995; Zhang et al., 1994). Four CCV‐B samples (two calcite and two aragonite) plot together with the carbonate phase samples of the sedimentary host rock in the field of near shore or restricted setting for seawater. This confirms the assumption that the first vein formation in the basalt and the lithification and cementation of Unit III sedimentary rocks are related. This happened probably near the ridge axis of the Cocos‐Nazca spreading center at shallow water conditions or in an area with restricted connection to an open ocean system (Figure 8a). Volcanological, geochemical, morphological, and geophysical data indicate that parts of the CCR formed under subaerial or shallow‐marine conditions (Hoernle et al., 2000; Werner et al., 1999). Compared to oxic and anoxic seawater (Bau et al., 1997), the elemental anomalies of the CCV‐B plot rather close to anoxic seawater and the anomalies of the CCV‐S closer to oxic seawater, except the Ce anomaly. The Y/Ho values of the CCV‐S plot in the field of seawater in open marine settings. This would imply that the formation of these veins happened off ridge axis somewhere on the way from the Cocos‐Nazca spreading center to the Middle America Trench (Figure 8b). However, strontium isotope ratios of the CCV‐S preclude pure seawater as fluid source and it should be taken into account that various parameters, such as pH, redox state, temperature of the system, and sorption along the pathway of the fluid control the REY exchange between the fluid and mineral (Debruyne et al., 2016).

The CCV‐B and CCV‐S shale normalized REE distribution patterns show HREE enrichment relative to LREE and MREE and positive La anomaly, which is typical for marine carbonates and indicating a derivation of the REE from seawater (Bolhar et al., 2004; Tostevin et al., 2016; Webb & Kamber, 2000; Zhang & Nozaki, 1996, 1998). The enrichment of HREE in the seawater is attributed to their different complexation behavior; where LREE and MREE are more preferentially adsorbed and HREE are preferentially remain in solution (e.g., Bolhar et al., 2004). Modern seawater is depleted in Ce, resulting in a negative Ce anomaly (e.g., Bolhar et al., 2004; Nozaki, 2001). Except for one sample (40R‐1‐W 6/9), a clear negative Ce anomaly is absent. The weak positive Ce anomaly values for CCV‐B and the lack of Fe‐oxyhydroxides and celadonite imply that the veins precipitated more likely under reduced conditions. Possible Ce mobilization during basalt alteration could result in Ce enrichment in the veins (Patino et al., 2003). The Europium anomaly is generally regarded as an indicator for hydrothermal input (e.g., Bolhar & Van Kranendonk, 2007; Wang et al., 2014; Wheat et al., 2002). Both CCV have slightly positive to positive values and indicate an influence of a hydrothermal fluid; this applies particularly to the CCV‐S and to the CCV‐B in highly altered basalt. The REY and isotopic values suggest that seawater and modified hot seawater are the main fluid source for both CCV. Fluid circulation occurred through both host rocks, resulting in a basaltic hydrothermal input in the CCV‐S and in the CCV‐B. The elemental and isotopic compositions of the CCV‐B confirm the results of previous analyses that carbonate precipitation occurred in multiple stages (Brandstätter et al., 2017).

### 5.2 Fluid‐Rock Interaction

The elemental composition of CCV can be strongly influenced by the chemical development of the fluid, by fluid‐rock interaction, as well as by alteration and leaching of elements from the host rock (e.g., Coggon et al., 2004). Certain major and trace elements, such as Mn, Fe, Mg, Na, and Sc, indicate fluid rock interaction in terms of leaching from the basaltic host and incorporation into the CCV‐B. This geochemical exchange is confirmed by chondritic Y/Ho values (26–28) for CCV‐B in highly altered basalt (Figure 7a). Elevated Mg, Mn, and Fe values for the calcite veins in the basalt, compared to the aragonite veins and CCV‐S, suggest element mobilization due to basalt alteration (e.g., partially altered to complete dissolved olivine; Bischoff & Dickson, 1975). At ODP Site 801 Alt and Teagle (2003) found highly altered basalts associated to deposits of low‐temperature hydrothermal fluids, indicating losses of Fe, Mn, Mg, Ca, Na, and Sr.

Elevated and scattered values for the lithophile elements Zr, Hf, and Th of CCV‐S samples indicate the imprint of the siliceous host rock (e.g., Bolhar & Van Kranendonk, 2007). Particularly sample 40R‐1‐W 6/9 shows strongly enriched values of Th, Hf, and Zr and anomalies differ compared to the other CCV‐S, which can be the result of contamination of the inhomogeneous sedimentary host rock during drilling of the >1‐cm‐thin calcite vein (e.g., Bolhar & Van Kranendonk, 2007). The U concentration of the sedimentary rocks can depend on the depositional and environmental conditions. Rapid sediment deposition limits U concentration due to slow diffusion rates below the sediment‐water interface (Wignall, 1994). Furthermore, under oxic conditions U is soluble; hence, under reducing conditions U may be more enriched (Algeo & Maynard, 2004). The U content is also strongly influenced by fluid‐rock interactions (Bach et al., 2011). A depletion in U is attributed to hydrothermal interaction of the fluid with the igneous host rock (Bach et al., 2011; Michard et al., 1983), which is also seen in our samples with lower U concentrations in the CCV‐B. Particularly the strontium isotopic composition of CCV indicates intense exchange of Sr between the fluid and the host rocks.

### 5.3 Thermal and Tectonic Evolution

First fibrous calcite and clay mineral vein formation in the CCR basalt occurred shortly after the basalt formation close to the Cocos‐Nazca spreading center. Veins were formed, due to temperature differences between the basalt and the seawater, resulting in thermal contraction and seawater invasion near or off‐axis (Figure 8a; Brandstätter et al., 2017). The similar trend of elemental and isotopic composition of some CCV‐B and the carbonate phase of the sedimentary host rock suggest that first CCV‐B precipitation was followed by sediment cementation and lithification from the same fluid source. The invaded seawater was heated up to 60 °C by the slowly cooling basaltic lavas. The aragonite veins show the highest ^87^Sr/^86^Sr ratios (~ 0.7087) and low formation temperatures. Due to their localized occurrence in the upper part of the igneous basement, the time of formation is not clear but could be around 17 Ma according to the strontium seawater curve.

Previous fluid inclusion studies showed a similar fluid chemistry for both CCV with a mean salinity of 2.4 mass % NaCl, and with 5.0 mass % NaCl for vesicles. The fluids yield temperatures up to 400 °C in CCV and quartz veins and nearly undeformed, intragranular fluid inclusions yield entrapment temperatures from approximately 70 to 200 °C in the CCV in both host rocks (Brandstätter et al., 2016). These temperatures are much higher compared to the calculated carbonate formation temperatures (from δ^18^O values) of the CCV‐S (60 °C to 90 °C) and CCV‐B (32 °C to 82 °C). Bach et al. (2011) described similar differences in temperatures of CCV in peridotite fault rocks. The isotopic and elemental characteristics imply that the CCV‐S and the transitional CCV‐B were precipitated from possibly 350 °C hot hydrothermal fluids, but the calculated formation temperatures, derived from δ^18^O values, were significantly lower. This is attributed to conductive cooling during slow ascent from deeper sources (Bach et al., 2011). In this study, the formation temperatures, derived from oxygen stable isotope compositions, present the precipitation temperatures of the carbonates, whereas the high temperatures of fluid entrapment in quartz precipitates may result from convective and/or advective heating of the fluid. All CCV, independent of the host rock, and the carbonate phase of the sedimentary host rock indicate in varying degrees hydrothermal influence, shown by a slight positive to positive Eu anomaly, which represents the imprint of the altered basalt and the exchange with the fluid. The stable and strontium isotopic compositions of the CCV suggest also the presence of a hot, CO_2_ rich fluid. This implies that after lithification of the sediments high temperatures, derived from a shallow magma chamber or intrusives, affected the CCR basalt and the sedimentary cover (Figure 8b). This could be the trigger of hydrofracturing in the lithified sedimentary rocks and subsequent vein formation, as well as precipitation of quartz in some CCV‐B (Brandstätter et al., 2016, 2017).

Brandstätter et al. (2016) discussed three potential heat sources for an additional heating event, (a) the Cocos‐Nazca spreading center, due to the ridge jump at 14.5 Ma; (b) a combination of Cocos‐Nazca spreading center and Galapagos hot spot activity; or (c) seamount intraplate volcanism in the area of Cocos Island in the Plio‐Pleistocene. Schindlbeck, Kutterolf, Freundt, Andrews, et al. (2016) cite a number of proposed models (Castillo et al., 1988; Harpp et al., 2005; Herbrich et al., 2015; O'Connor et al., 2007; Werner et al., 2003) to explain such young volcanism along the CCR, and the models coincide that Cocos Island Seamount Province volcanism has its origin from plume‐related mantle movements. The special location of the Cocos Island at the eastern edge of an extinct small spreading center and the delimitation from the thickened lithosphere of the CCR by a transform fault could be the reason for the significant larger melt catchment area of Cocos Island compared to adjacent seamounts farther away from the transform fault (Schindlbeck, Kutterolf, Freundt, Andrews, et al., 2016). Herbrich et al. (2015) propose that contrary to the CCR the Cocos Plate Seamount province has its origin on EPR generated crust/lithosphere. The formation of the seamounts is the result of northward transport of plume material from the Northern Galapagos area and incorporation of depleted asthenosphere material. By reaching the thinner EPR lithosphere, melting occurs due to decompression or is the result of upwelling at the edge of a viscous relict root of accumulated plume material at the base of the lithosphere (Herbrich et al., 2015). The models of Schindlbeck, Kutterolf, Freundt, Andrews, et al. (2016) and Herbrich et al. (2015) endorse that there is a melt catchment area between the Galapagos hot spot and Cocos Island with lateral distribution towards the lithosphere of the CCR.

The elemental data presented in this study, particularly the high Y/Ho ratios for the CCV‐S, and the tephra age in Unit III, suggest rather intraplate seamount volcanism northward of the Galapagos hot spot as possible heat source, than the Cocos‐Nazca spreading center. Due to fast plate tectonic movement of 96 mm/a (>11 Ma) to 72 mm/a (<10 Ma), Hole 344‐U1414A was between the Cocos Island and the spreading center 12 Ma ago (Schindlbeck et al., 2015) and the subsequent younger magmatic processes of the Cocos Island Seamount Province affected Hole 344‐U1414A. While approaching the Middle America Trench cooling was accompanied by deformation due to bending of the Cocos Plate (8c). Microstructural analysis of CCV obtained deformation temperatures <220 °C in the CCV‐S (Brandstätter et al., 2017).

## 6 Conclusion

The differences between the CCV‐S and the CCV‐B are most pronounced in their REY patterns, especially Y/Ho ratios, Mn, Fe, Sc, Na, Zr, Hf, Th, and U content, in their Mg/Ca ratios, oxygen isotopic composition and in their ^87^Sr/^86^Sr ratios. These differences are mainly attributed to different fluid sources and to the corresponding host rock and the interaction with the fluid. The isotopic and elemental composition of aragonite and fibrous calcite veins in the CCR basalt indicate that invading seawater from restricted marine setting was the fluid source and/or precipitation occurred under restricted conditions. The chemical composition of the CCV‐B in highly altered basalt corresponds rather to the geochemistry of the basaltic host rock and to a hydrothermal fluid, than to seawater. The high Y/Ho ratios and positive La and Gd anomalies suggest that CCV‐S were precipitated from seawater of more likely open marine setting or from a fluid chemical changed by fluid‐host rock interaction. The positive Eu anomaly indicates that all CCV were hydrothermally influenced and the strontium and oxygen isotopic composition of the CCV‐S and of the composite CCV‐B samples imply that seawater was modified into a hot, hydrothermal fluid. Fibrous calcite veins in the basalt were precipitated from invaded seawater near the ridge axis. These veins in the CCR basalt, as well as lithified sedimentary rocks, were penetrated by a hot and chemically modified fluid, resulting in vein formation in the sedimentary rocks and quartz and calcite precipitation in the CCV‐B. The lack of high‐temperature alteration in the basalt could be attributed to a high fluid flow rate, evidenced by hydrofracturing in the sedimentary rocks of Unit III. The geochemical results combined with the age data of Unit III and the models for formation of the Cocos Plate Seamounts after Herbrich et al. (2015) and Schindlbeck, Kutterolf, Freundt, Andrews, et al. (2016) constrain intraplate seamount volcanism in the area between the Galapagos hot spot and the Cocos Island, probably in the Plio‐Pleistocene, as the source for subsequent heating after CCV formation.

## Supporting information

Supporting Information S1

Data Set S1
