# Supplementary material for: The Origin of Carbonate Veins Within the Sedimentary Cover and Igneous Rocks of the Cocos Ridge: Results From IODP Hole U1414A
Source: Geochem Geophys Geosyst. 2018 Oct 11;19(10):3721–38. doi: 10.1029/2018GC007729 (PMC6282762; doi:10.1029/2018GC007729)
Supplement: Supplementary file 1 — Supporting Information S1 [file GGGE-19-3721-s001.docx]

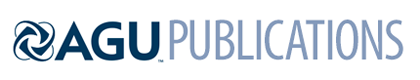


*Geochemistry, Geophysics, Geosystems*

Supporting Information for

**The origin of carbonate veins within the sedimentary cover and igneous rocks of the Cocos Ridge: Results from IODP Hole U1414A**

Jennifer Brandstätter^1^, Walter Kurz^1^, Sylvain Richoz^1,2,^ Matthew J. Cooper^3^ and Damon A.H. Teagle^3^

1Institute of Earth Sciences, NAWI Graz Geocenter, University of Graz, Graz, Austria,

2Department of Geology, Lund University, Lund, Sweden,

3Ocean and Earth Science, National Oceanography Centre Southampton, University of Southampton, SO14 3ZH, England, UK

**Contents of this file**

Tables S1

**Additional Supporting Information (Files uploaded separately)**

Captions for Tables S1

**Introduction**

Supplementary material includes data table for major, and trace element analyses of the strongly lithified calcareous and siliceous cemented sedimentary host rock, the additionally extracted carbonate phase of Unit III and of the Cocos Ridge basalt of IODP Hole 344-U1414A.

Table S1. Is large and uploaded separately and includes the elemental composition, calculated anomalies and ratios of the host rock sample material of Unit III and of the Cocos Ridge basalt.
